# Colloidal stability and dielectric behavior of eco-friendly synthesized zinc oxide nanostructures from Moringa seeds

**DOI:** 10.1038/s41598-024-52093-5

**Published:** 2024-01-28

**Authors:** Basílio José Augusto José, Mahendra Devidas Shinde

**Affiliations:** 1 Faculty of Science and Technology, Licungo University, Beira, Mozambique; 2 School of Science, Sandip University, Nashik, India; 3 School of Engineering and Technology, Sandip University, Nashik, India

**Keywords:** Materials science, Nanoscience and technology

## Abstract

This study centers on the environmentally benign synthesis of zinc oxide nanoparticles (ZnO NPs) derived from Zn (CH_3_COO)_2_·2H_2_O and Moringa seeds. The synthesized nanostructures underwent comprehensive characterization utilizing diverse analytical techniques, encompassing X-ray diffraction (XRD), UV–VIS spectroscopy, field-emission scanning electron microscopy (FESEM), X-ray photoelectron spectroscopy (XPS), and Raman spectroscopy. XRD measurements coupled with W–H plot transformation unequivocally confirmed the formation of ZnO nanostructures, characterized by an average size of 24.9 nm. UV–VIS spectroscopy, complemented by Kubelka Munk curve analysis, elucidated the direct conduction and determined a bandgap of 3.265 eV. FESEM analysis revealed minimal particle aggregation, showcasing well-defined grain boundaries spanning sizes from 20.4 to 87.7 nm. XPS analysis substantiated the presence of Zn (2p), Zn (3p), Zn (3d), and O (1s). Raman spectroscopy identified E_2_H as the predominant mode, followed by E_1_(TO) and (E_2_H-E_2_L). ZnO thin films, fabricated via pulsed laser deposition (PLD) and deposited onto silicon (100) substrates, exhibited exemplary morphology and discernible topography, characterized by a normal grain size distribution. Zeta potential tests yielded a value of approximately ($\mathcalligra{z}$ ~ − 43.8 mV), indicative of the commendable stability of the colloidal suspension, likely attributable to low particle aggregation. Dielectric measurements conducted on sintered pellets at 900 °C unveiled elevated capacitance and dielectric constant at low frequencies across the temperature range of 289.935–310 K. These findings affirm the potential utility of environmentally synthesized ZnO for a spectrum of applications, including energy devices and nanofluids.

## Introduction

The progression of environmentally conscious materials has become a focal point of considerable interest within the scientific realms of nanotechnology and materials science. This heightened attention is particularly pertinent in the contemporary era, marked by the pervasive consequences of climate change and global warming. These far-reaching challenges have been extensively deliberated upon in prominent international forums, such as the recent COP27 conference held in Egypt. Furthermore, the significance of environmentally conscious materials aligns with the broader objectives outlined in the sustainable development goals (SDG 7, 11, and 13)^1–3^. Approaches aimed at creating such materials are often referred to as "green" when they involve the utilization of active components derived from natural sources to reduce toxicity levels, or as "eco-friendly" when there is a focus on balancing product quality with minimizing environmental impact during the synthesis process^4–10^.

Zinc oxide (ZnO) exhibits remarkable versatility and finds applications in a wide range of fields, including energy devices^11^, biomedical applications^12–14^, and sensing^15–17^.

As Punica belongs to polyphenols group, their reaction with Zinc ions forms complex molecules of Zn (OH)_2_ by capping and reducing process which are turned into respective nanoparticles during annealing process at around 500 °C. In the pursuit of cultivating uniform thin films with high crystallinity quality on p-Si (100) for photovoltaic applications, a methodology involving the spin coating technique was employed. The thin films were fashioned using ZnO nanoparticles (NPs) derived from Punica granatum (pomegranate) juice extract, with a green synthesis approach, as detailed in reference^18^. The prepared films exhibited wurtzite hexagonal arrangement of 60 nm in crystallite size, 3.41 eV of direct bandgap by UV–VIS spectroscopy, distributed in two components Zn and O from EDX analysis. The study was not extended to dielectric measurement in order to confirm the potential application of the films into light-emitting diodes apparatus.

In recent studies, zinc oxide has been synthesized utilizing various components of the *Moringa oleifera* plant, including the leaves^19,20^ root extract^21^ and seeds^22^.

Beyond the reported findings, the crystal structures of wurtzite (hexagonal) and zincite, along with their direct band gap ranging from 3.0 to 3.7 eV in pure form, high transparency, and adaptability, provide green synthesized ZnO NPs with potential applications in energy devices and storage^11^ (thin films), enhancement of cement concrete properties^23–29^ (powder and nanofluid), water purification^30–33^ (nanofluids and thin films), engine coolants^34^, EOR^35,36^ (nano fluids) and gas sensors.

In green synthesis the plant extract plays different roles. As reducing agent, the active components of the green material in aqueous solution convert metal ions into their respective nanoparticles. As stabilizer, they act as surfactant to control the dispersity of nanoparticles minimizing their agglomeration. When extracts act as capping agents, nucleation by aggregation takes place forming small clusters, where the designing of the shape and size is performed. During this process Ostwald ripening might occur, where larger particles grow at the expense of smaller particles through the diffusion of atoms Azeez et al.^37–42^.

Moringa, also known as the Horseradish tree, Mulangay, Mlonge, Benzolive, Drumstick tree, Sajna, Kelor, Saijihan, and Marango, is an indigenous tree that belongs to the Moringaceae family. It is naturally found in Africa, Arabia, South and East Asia, the Pacific and Caribbean islands, and South America^43^. This tree is renowned for its nutritional and medicinal benefits.

Moringa boasts a rich array of phytochemicals, showcasing vital vitamins such as l-ascorbic acid (vitamin C), retinol (vitamin A), and niacin (vitamin B3). Furthermore, it encompasses an impressive variety of flavonoids, including quercetin, kaempferol, myricetin, and isorhamnetin, as well as notable phenolic acids like ellagic acid, gallic acid, chlorogenic acid, and caffeic acid. This diverse composition divided into 5 groups (Table 1) underscores the nutritional value and potential health benefits of Moringa and contribute to the formation of ZnO NPs^21,44^.

**Table 1:** The comprehensive composition and individual roles of Moringa seed components^19,45–49^.

N°	Phytochemicals	Role during synthesis of ZnO	Mechanism of formation
1	Phytosterols	Stabilizers of zinc ions	Regulating particle size, morphology, and structure
2	Flavonoids	Reduction of zinc ions	Reduce zinc ions, transforming them into nanoparticles while mitigating undesired side reactions during synthesis
3	Polyphenols	Reducing and stabilizers of Zn^2+^	Coat the surface of nanoparticles to prevent clustering and facilitate the reduction of Zn^2+^
4	Amino acids and proteins	Capping and stabilizer of Zn^2+^	Incorporate into the ZnO surface to prevent the formation of clusters, simultaneously providing stabilization and reduction
5	Lipids	Hydrophobicity of ZnO NPs	Mult-compatibility and wettability through interaction with the ZnO surface

### Different approaches of synthesizing zinc oxide nanoparticles

In the green synthesis process, the phytochemicals present in moringa interact with zinc ions, leading to the formation of zinc oxide (ZnO) nanoparticles. These synthesized ZnO nanoparticles hold great potential for applications in biomedicine and energy storage technology^48^.

**Figure 1:**
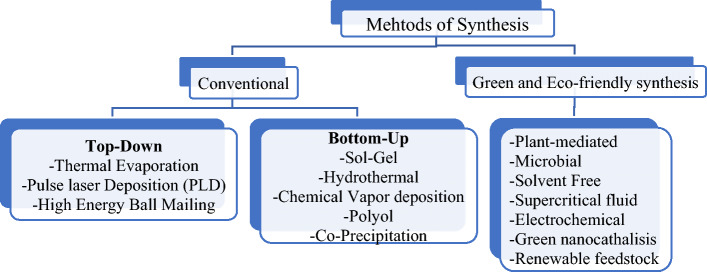
Methods of synthesis.

In the existing literature, various conventional synthesis methods^50^, as well as green and eco-friendly synthesis approaches^37,51–53^ have been reported. These methods are depicted in Fig. 1.

In this experiment, special attention is given to plant-mediated synthesis of ZnO nanoparticles (NPs) due to its numerous advantages. Plant-mediated synthesis is considered environmentally friendly as it reduces the need for excessive chemical usage. It is also cost-effective, requiring fewer chemicals and materials. Furthermore, this method has the advantage of low toxicity as it involves the use of smaller quantities of potentially harmful substances. The reliability of the plant-mediated synthesis method is another advantage, as it can be conducted in a single pot, making it convenient and efficient^38,48,54^.

The properties of ZnO NPs significantly impact their behavior in colloidal suspension. This behavior can be observed through their stabilization, coagulation, or flocculation in a fluid medium. The measurement of zeta potential and particle size analyzer is used to study and evaluate this behavior, and the corresponding parameters are presented in (Table 2).

**Table 2:** Zeta potential values of colloidal suspension^55^.

Zeta potential (mV)	Stability behavior of the colloids
From 0 to ± 5	Rapid coagulation and flocculation
From ± 10 to ± 30	Insipient instability
From ± 30 to ± 40	Moderate stability
From ± 40 to ± 60	Good stability
More than ± 61	Excellent stability

The effect of water and ethylene glycol as surfactants, along with sodium decency sulfate (SDS), cetyltrimethylammonium bromide (CTAB), and sodium carboxymethyl cellulose as adsorbents, was investigated in a study^56^. It was observed that at a pH of 7.3 and a starting titration diameter size of 50 nm, the particles exhibited a positive zeta potential of + 35 mV. Furthermore, the stable region of the particles was extended by the addition of cationic surfactants, resulting in a zeta potential of 30 mV at neutral pH. However, according to the literature, these particles were found to have moderate stability and were deemed unsuitable for use as nanofluids.

In another investigation^57^, ZnO nanofluids were found to have a stability of − 14 mV, while surface-modified ZnO showed a stability head of − 45.4 mV.

The dielectric properties of 15 nm ZnO nanoparticles synthesized through the co-precipitation method were studied^58^. It was observed that these nanoparticles exhibited a high dielectric constant and dielectric loss at low frequencies, which could be attributed to the formation of boundaries between particles. Additionally, the electrical conductivity of the nanoparticles improved within the temperature range of 290–409 K, which was attributed to the presence of defect centers and charge carriers.

Electrical properties of ZnO nanoparticles with a size smaller than 50 nm, synthesized via the sol–gel method and sintered at 1050 °C, were investigated^59^. It was observed that the I–V curve exhibited nonlinearity, characterized by a breakdown voltage, non-linear coefficient, and leakage current, which were influenced by the particle size and impurities. Furthermore, an increase in temperature was found to enhance the electrical properties of the material.

The effect of different solvents on the I–V behavior of ZnO was investigated in previous studies^60,61^. The experiments compared the behavior of ZnO in a solvent-free environment and in the presence of 2-propanol solvent. Both conditions exhibited good current intensity, while the nonlinearity behavior remained consistent in both the dark and under UV light, with a slight improvement observed in the latter.

In a separate investigation focusing on solid-state battery applications, dielectric measurements were performed on ZnO doped with Co. The results indicated that the sample containing 1.5 wt% ZnO/Co nanoparticles demonstrated an improvement in ionic conductivity^62^.

While numerous published articles on the green synthesis by Moringa have primarily focused on the extracts from leaves^19,21,22,48^, studies unveiled a wealth of nutrients, strong surface hydrophobicity, foaming agent, and notable potential for water purification in Moringa seeds^46,47,49^ in comparison to leaves.

Despite the abundance of studies on Moringa, there is a notable dearth of research specifically addressing the colloidal stability of Moringa seeds^46^ and dielectric behavior of ZnO prepared using these seeds. This research endeavors to fill this gap by exploring the inherent potentials of Moringa seeds in the synthesis of ZnO nanoparticles from [Zn (CH_3_COO)_2_·2H_2_O]. Furthermore, the study aims to elucidate their influence on colloidal stability and dielectric behavior, shedding light on the multifaceted applications of Moringa seeds in nanotechnology and material science.

The properties of these nanostructures were comprehensively characterized, including their structural, surface, optical, electrochemical, and dielectric characteristics, with the aim of understanding their potential applications in energy devices and nanofluids^19,21,22,48^.

## Materials and methods

### Materials

For the synthesis was used: 10.97 g of [Zn (CH_3_COO)_2_·2H_2_O] from Merck, 6 g of MSP, 0.7 g of NaOH, 400 ml DW, 100 ml of CH_3_CH_2_OH, Centrifuge, Probe sonicator, Oven and Muffle Furnace.

### Preparation of Moringa seeds powder (MSP)

**Figure 2:**
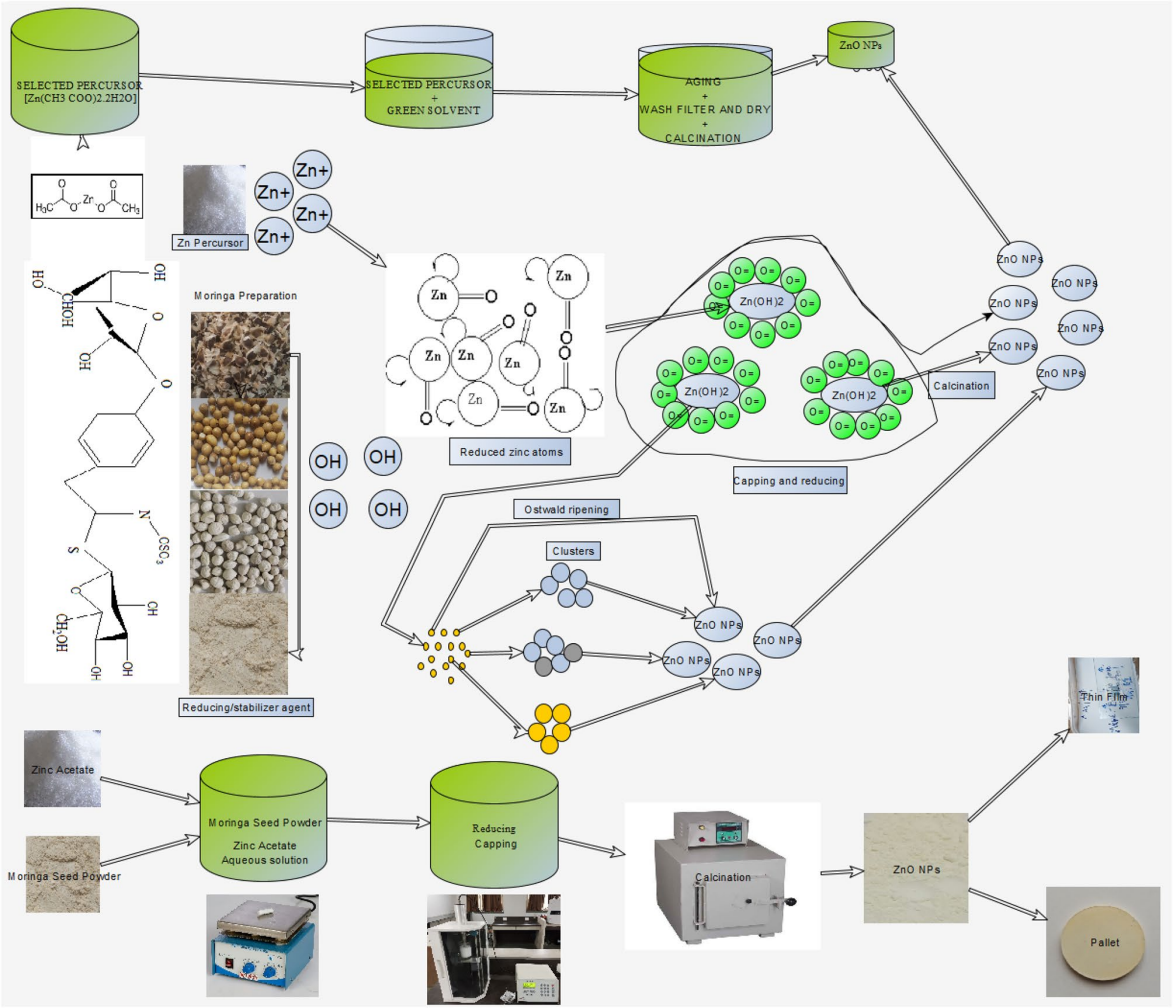
Mechanism of formation of ZnO from Moringa seeds.

**Figure 3:**
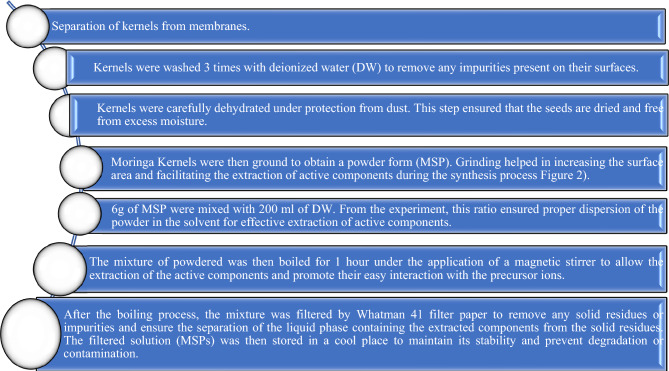
Preparation of Moringa seed powder for synthesis.

Moringa seeds (Fig. 2), sourced from Nashik, underwent the subsequent processing steps shown in Fig. 3, following all methods in accordance with the relevant guidelines^63,64^.

### Synthesis of ZnO NPs, thin films coating and pallets preparation

Synthesis was carried out by mixing 10.97 g of [Zn (CH_3_COO)_2_·2H_2_O] with 100 ml of MSPs solution and stirring the mixture at 60 °C for 1 h. Sequentially, 5 pallets of NaOH were added. The combination of MSPs (Fig. 2) and 0.7 g of NaOH at 60 °C accelerated the nucleation, capping, and Ostwald Ripening formation of nanoparticles. The solution was then subjected to probe sonication for 15 min (Fig. 2). Subsequently, the solution was allowed to precipitate for 3 h, and the supernatant was separated from precipitate by using a syringe. The remaining white solution was transferred to test tubes and centrifuged at 3500 RPM for 5 min^38^. Once again, the transparent solution and the remaining white solution were transferred to a new beaker for washing with CH_3_CH_2_OH and DW. After washing, the precipitant was removed using a syringe, and the precipitate material was transferred to a crucible and dried in an oven at 80 °C for 48 h. After complete drying, the obtained powder was subjected to calcination at 500 °C for 2 h to remove impurities and form ZnO^65^. Due to particle aggregation, the formed ZnO was ground multiple times using a mortar and pestle to obtain a fine powder (NPs), which was then stored in a 20 ml brown glass bottle for characterization and further treatment.

To prepare thin films and dielectric measurement, quantities of 0.6 g and 2 g of ZnO NPs were used to prepare two pallets of 2 mm in thickness, a radius of 8 mm and 25 mm, respectively (Fig. 2). The 25 mm was used as a target for thin film deposition in pulse laser deposition (PLD) while the 8 mm pallet used for dielectric measurement. The pallets were compressed under a force of 50KN for 5 min to solidify them. Subsequently, they were sintered at 900 °C for 5 h, with a temperature increment rate of 5 °C per minute^66^. Sintering is a heat treatment process used to fuse the particles together and increase the density and strength of the ZnO pallets.

The film was deposited in p-Si (100) substrate at 700 °C for 30 min under a pressure of 1mb (0.76 Torr) inside the chamber. Annealing was performed at 500 °C for 1 h under an oxygen atmosphere to reduce defects and vacancies inside the chamber. The laser parameters for the deposition process were set as energy per pulse of 317 nJ, pulse frequency of 5 Hz, chamber pressure of 3463 mbar, and laser power of 1.58 W.

To perform the dielectric measurement, the resistance of the pallet was measured using a multimeter and found to be approximately 53 MΩ. The pallet was then polished using emery paper until a mirror surface was achieved. It was further cleaned with acetone to remove any impurities. The sample was coated with pure silver to enable conductivity and create a parallel plate capacitor geometry. The prepared sample was inserted into the dielectric probe, with the bottom conducting part of the probe consisting of a plate and the upper conducting part made of copper wire soldered onto the silver-coated sample.

The bottom part of the probe was connected to high voltage and current sources, while the upper side was connected to low voltage and current sources. Additionally, a ground connection was established to remove any leakage currents. The measurement was conducted under high vacuum conditions after adjusting parameters such as frequency and temperature (“Dielectric properties of the ZnO”).

### Characterization techniques

**Figure 4:**
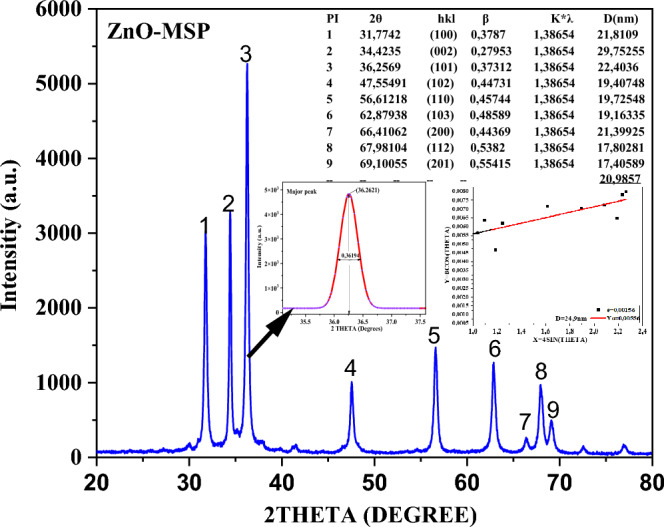
XRD diffraction patterns of ZnO.

The powder and thin films were subjected to structurally characterization: *XRD:* d8 Advanced 40 kV, 2.2KW sealed X-ray tube (Cu Kα), LynxEye 1D-PSD, surficial: *Field Emission Scanning Electronic Microscopic* (FEI NOVA NANOSEM 450 FE-SEM-RV), *Atomic Force Microscope* (AFM-Zeiss LSM510META). Electrochemical: Zeta potential (Nanoplus 3 GSO), spectroscopically: *UV–VIS Spectrophotometer* Vertex 70, *Raman spectroscopy.* HORIBA Jobin LabRAM HR800, which was coupled with an OLYMPUS BX41 microscope, *X-Ray Photoelectron spectroscopy* (ESCA spectrometer is SPECS Surface Nano Analysis GmbH, Germany made, Al K-alpha 1486.61 eV) and dielectric measurement (dielectric and PE loop RR) for the pallet of 2 × 8 mm (Fig. 4).

## Results and discussions

### XRD

**Figure 5:**
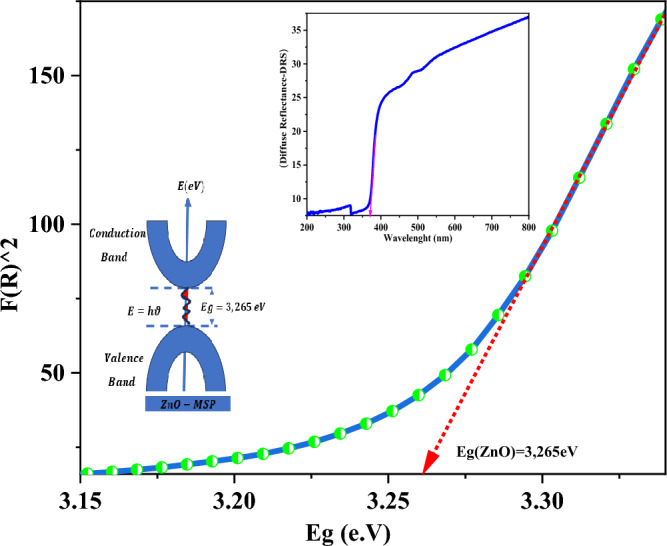
Schematic diagram of UV VIS spectroscopoy and energy gap of ZnO.

The structural characterization of ZnO was performed using X-ray diffraction (XRD) in the range of 20°–80° (Fig. 5). The obtained peaks were in accordance with the reference (JCPDS card No 75-1621)^67^ indicating that the ZnO is hexagonal-wurtzite structure.

As observed from the refined and fitted scattering pattern of the peaks, the average crystallite size was determined to be 20.9857 nm using the Debye–Scherrer formula. However, when applying the Williamson–Hall Plot, the average crystallite size was calculated as 24.9 nm (Fig. 5). The slight difference between these two values can be attributed to the accuracy of the Williamson–Hall Plot, which takes into account macrostrain and β, whereas the Scherrer equation relies solely on the values of β for calculation^68^.

Similar patterns were obtained in both conventional synthesis (chemical-based)^69,70^, green and eco-friendly synthesis^38,65^ for pure and modified ZnO. The promising aspect of these consistent findings across different approaches is that they demonstrate the potential to reduce the reliance on chemicals by utilizing the phytochemical actives present in Moringa seeds.

### UV–VIS spectroscopy

**Figure 6:**
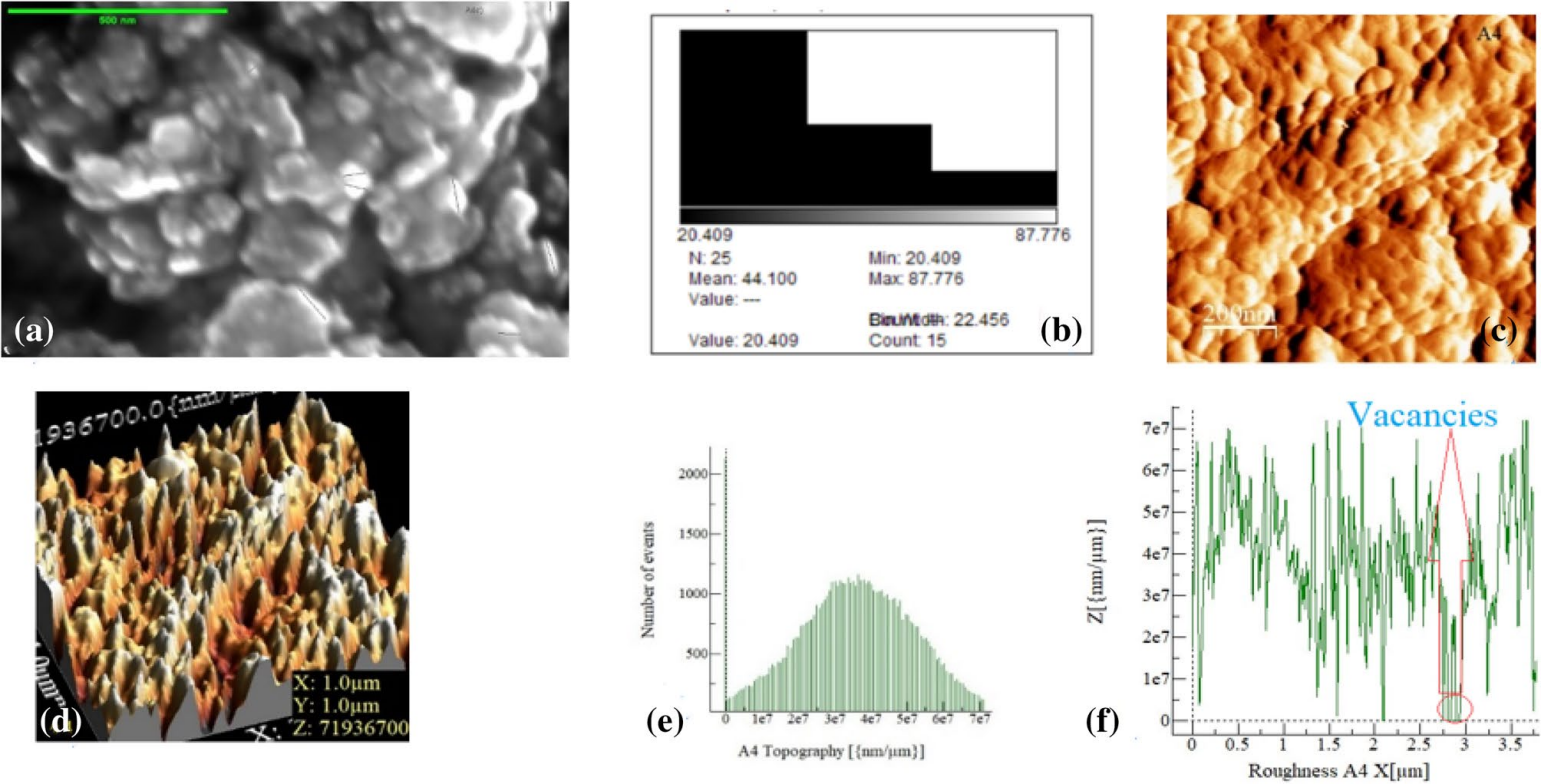
2D, 3D view of FESEM and AFM of ZnO synthesized from Moringa seeds.

The UV–Vis spectroscopy measurement was used to determine the energy gap and conduction type of the ZnO NPs. To correlate the diffuse reflectance data with the absorption properties of the material (Fig. 6), a mathematical procedure known as Kubelka–Munk was employed. This procedure involves five steps: (a) converting the diffuse reflectance data (R) to a percentage $R=\frac{\%R}{100}$, (b) calculating the molar absorption coefficient $K={(1-R)}^{2}$, (c) determining the scattering factor $S=2R,$ (d) calculating the Kubelka–Munk function $F\left(R\right)=\frac{K}{S}$, $F{(R)}^{2}$ (e) calculating the energy ($E=\frac{h\cdot c}{\lambda }$) using the wavelength (λ) values given in the diffuse reflectance spectra.

The energy gap determined by this procedure was found to be $3.265 {\text{eV}}$ (Fig. 6), which falls within the range of the literature-reported energy gap for pure ZnO^41^. The upward position and the steepness of the Kubelka–Munk function is related to a direct band gap. In this mechanism of conduction, the minimum energy in the conduction band aligns with the maximum energy in the valence band. Electron transitions between the valence and conduction bands occur through the absorption or emission of photons (light). This type of conduction is characterized by high absorbance and a low scattering coefficient, making these materials suitable for applications such as LEDs, lasers, and solar cells.

### FESEM and AFM

To gain further insights into the properties of the synthesized ZnO, both powder and thin films were evaluated. Figure 6a,c show 2-D field emission scanning electron microscopy (FESEM) and c) atomic force microscopy (AFM) images, respectively, captured at the nanometric scale. The structure of the ZnO reveals polydispersity, solid-filled grains and aggregates with sizes ranging from 20.409 to 87.776 nm, as observed in Fig. 6b. These findings correlate with the observations made in Fig. 4, which illustrates the XRD patterns in terms of size.

Figure 6d provides a 3D view of the surface image scanned in a (1 μm × 1 μm) area of the ZnO deposited on a p-Si (1000) substrate using pulse laser deposition (PLD). The topography of the deposited particles is observed to be normally distributed, as shown in Fig. 6e characterized by roughness with the presence of hills and hollows, as depicted in Fig. 6f. This roughness may have an impact on the analysis of transport phenomena, including (I–V) measurements.

### XPS

The elementary composition, chemical state analysis, and surface characterization of the ZnO nanostructures were examined using X-ray photoelectron spectroscopy (XPS) with Al K-alpha (1486.61 eV) X-rays generated by a SPECS Surface Nano Analysis GmbH instrument in the energy range of 0–1300 eV. The XPS measurements were conducted with an acceleration voltage of 13 kV and a power of 100 W. XPS analysis provides valuable information about the elemental composition, oxidation states, and chemical bonding of the surface species present in the ZnO nanostructures^71^.

**Figure 7:**
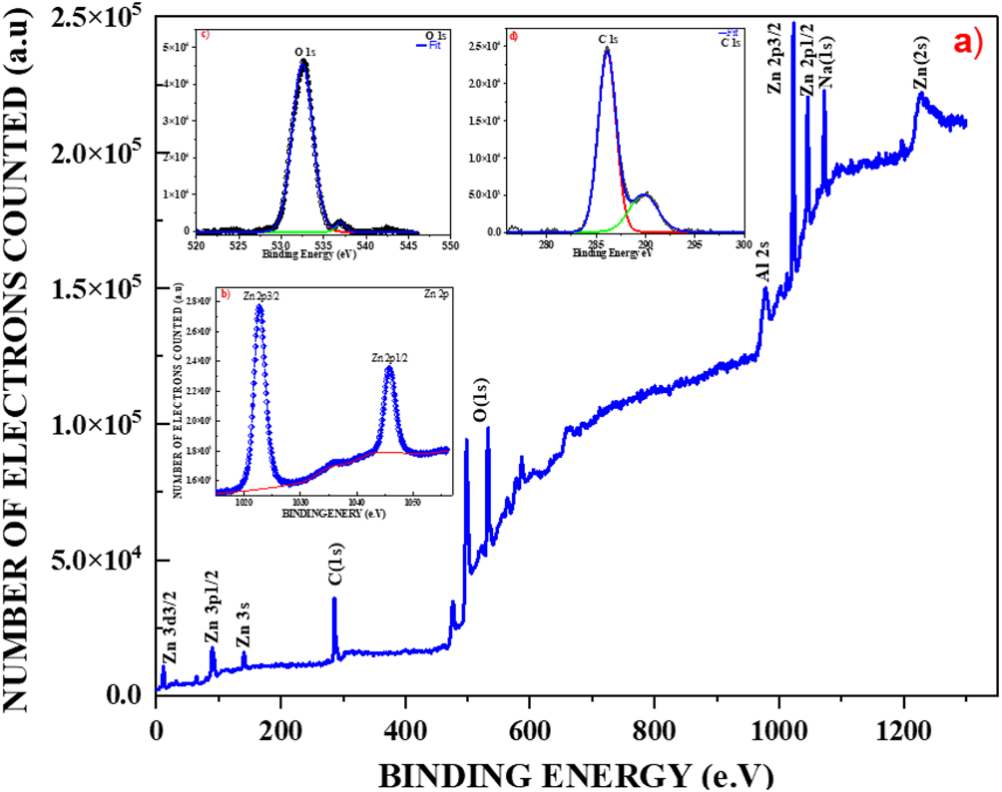
XPS analysis of ZnO from Moringa seeds.

The experimental data related to Zinc were compared with theoretical data given by Lebugle et al.^72^, who reported the experimental L and M core levels' binding energy for the metals ^22^Ti to ^30^Zn. Some slight differences, less than 0.1%, were observed during the comparison of the experimental and reference data given in Fig. 7a. This discrepancy could be attributed to differences in the measuring instruments used, as the reference data used the Hewlett-Packard 5950 A ESCA. Another possible reason could be the different approaches employed. The reference data utilized chemical synthesis, which was conducted in 1981, prior to the green synthesis approach.

Two intense peaks located at 1022.5 eV and 1045.5 eV were observed, which are attributed to the Zn 2p_3/2_ (1022, 0 eV) and Zn 2p_1/2_ (1045, 1 eV), respectively, corresponding to Zn–O bonding within the hexagonal Wurtzite structure^73,74^. These reference data are reported in the core-electron binding energies of the first thirty elements. Additionally, Zn was observed at 9.8 eV, with a reference value of (Zn 3d_3/2_ 9.77 eV), 91.5 eV with a reference value of (Zn 3p_1/2_ 91.4 eV), 142.5 eV with a reference value of (Zn 3s 142 eV), and Zn (2s) (1206.00)^75^.

In the spectrum, several other elements were identified, including 285.3 eV as (C 1s 285.0 ± 0, 3)^76^ for pure native elements, 535.2 eV as (O1s 532 eV), and 956.7 eV that might be related (Al 2s). Additionally, the peak at 1071.1 eV might be related to Na (1s) (1071, 7 ± 0, 7)^71,75,77–79^.

In their meticulous experimental research^80^ explored the utilization of zinc and gallium oxide for nanoparticle synthesis, emphasizing eco-friendly methodologies. Table 3 show the comparison between their findings and the experimental data of this research demonstrating that these approaches offer a viable solution for producing high-quality nanoparticles that are environmentally friendly.

**Table 3:** Comparison of BE between eco-friendly and chemical synthesis^80^.

Peaks found	Our experimental BE (eV) ZnO-MSP eco-friendly synthesis (eV)	Schon^80^
Zn 3d_3/2_	9.84	Zn 3d (10.1 ± 0.2 eV)
Zn 3p_1/2_	91.5	Zn 3p_1/2_ (91.4 ± 0.3 eV)
Zn (3s)	142.5	Zn (3s) (139.6 ± 0.3 eV)
C (1s)	285.3	C(1s) (285.1 ± 0.2 eV)
O (1s)	535.2	O(1s) (530.6 ± 0.2 eV)
Zn 2p_3/2_	1022.5	Zn 2p_3/2_ (1021.7 ± 0.2 eV)
Zn 2p_1/2_	1045.5	Zn 2p_1/2_ (1044.8 ± 0.2 eV)
Zn (2s)	1210.1	Zn(2s) (1195.9 ± 0.3 eV)

The presence of deconvoluted major peaks of Zn 2P is shown in Fig. 7b. The fitted binding energy of oxygen is presented in Fig. 7c, while the fitted graph related to carbon is shown in Fig. 7d.

### Raman spectroscopy

To gain a deeper understanding of the energy excitation modes of ZnO, Raman spectroscopy analysis was conducted. The measurement was performed using the HORIBA Jobin LabRAM HR800 instrument, equipped with an OLYMPUS BX41 microscope and a 473 nm laser. The Raman shift values obtained from the analysis were used to identify specific vibrational modes and characterize the crystal structure of the ZnO nanoparticles. This was achieved by comparing the observed Raman peaks with reference spectra and known vibrational modes of ZnO.

ZnO wurtzite structure is part of space group ${C}_{6v}^{4}$ and the theory predicted the existence different Raman modes^81^.

$$\Gamma ={A}_{1}+2{B}_{1}+{E}_{1}+2{E}_{2}$$

where $({A}_{1}and {E}_{1})$ are polar and split into transverse optical ${A}_{1}(TO)and {E}_{1}(TO)$ and longitudinal ${A}_{1}(LO)and {E}_{1}(LO)$ components. In the same way, ${E}_{2}$ consists of two modes (${E}_{2}H$ and ${E}_{2}L$). The presence of ${E}_{2}$ is associated to vibration of Zinc or oxygen also impurities and defects^81,82^.

**Figure 8:**
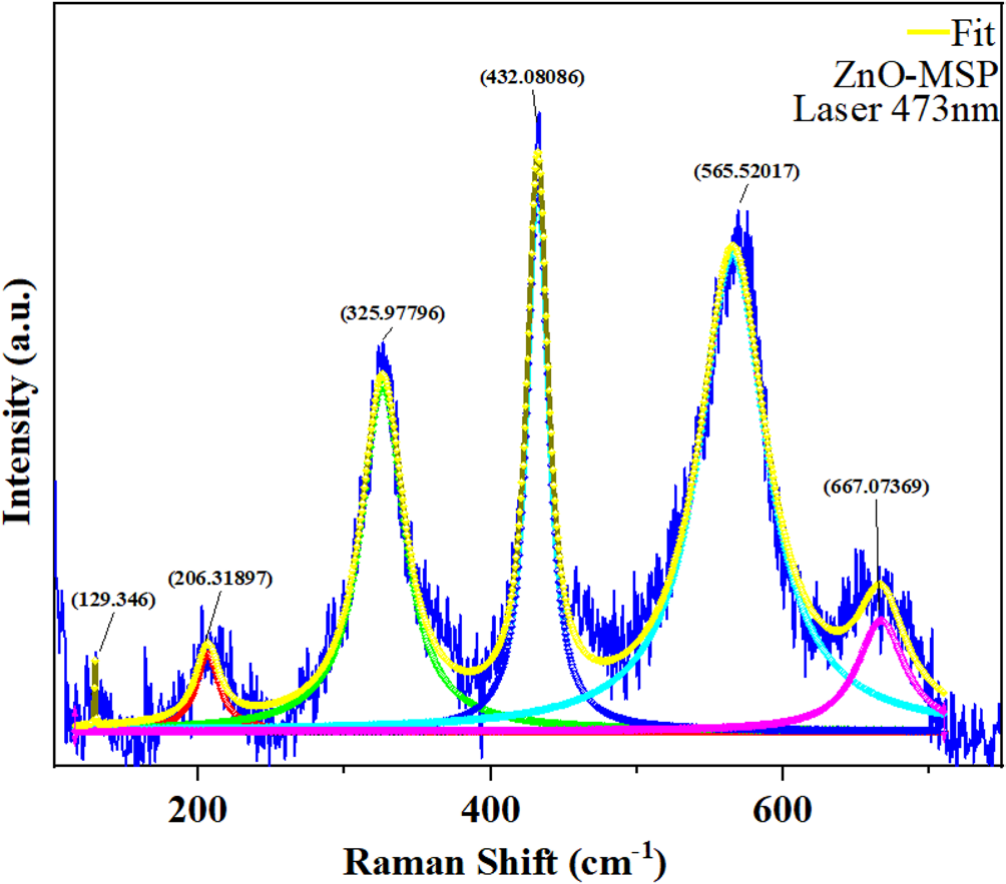
Raman spectroscopy of ZnO in the range of 100–700 cm^−1^.

Figure 8 presents the comprehensive Raman spectra of ZnO-MSP, covering the range between100 and 750 cm^−1^. In order to determine specific values such as Raman intensity and Fourier coefficient, the phonon confinement mode (PCM) method is commonly utilized^83^. The given peaks are 129,346 cm^−1^, 206,31,897 cm^−1^, 325,97,796 cm^−1^, 432,08,086 cm^−1^, 565,52,015 cm^−1^ and 667,0769 cm^−1^. The appearance of 129,346 cm^−1^ (${E}_{2}^{Low}$) in the low wave range (LWR) is considered as Raman active Branch for ZnO, due to vibration of oxygen atoms, similar explanation given to 206,31,897 cm^−1^ represented by 2TA, 2E_2_(LO)^82^. The presence of 326,9 cm^−1^ peak, might be related to (E_2_H–E_2_L) due to the longitudinal optical (LO) phonon mode that also involve vibration of oxygen atoms within the crystal lattice^82^. Additionally, E_2_H mode of 432,08086 cm^−1^ in the high wave range (HWR) is the dominant peak and is attributed to transversal optical phonon mode which involve vibration of oxygen atoms perpendicularly to crystal axis^84^. Based on the experiment conducted with ZnO nanoparticles having a crystallite size of 25 nm, it was observed that the E2 band in the Raman spectra represents a characteristic band associated with the wurtzite phase. The appearance of 569,22 cm^−1^ related to A1 (LO) longitudinal optical phonon mode involving vibration of Zinc and oxygen atoms in the crystal lattice^82^. Finally, the presence of 569,22 cm^−1^, E_1_ (TO) is attributed to multiple phonons scattering process of ZnO^85^.

### Zeta potential

Zinc oxide was investigated in terms of its colloidal stability in a fluid medium, which is one of the determinants of its potential applications as a nanofluid. The stability of the particles was evaluated by measuring the zeta potential. ZnO nanofluids are known for their wide range of applications in biomedicine, engine coolants, enhanced oil recovery (EOR), and water treatment, as mentioned in references^35,40,86–89^. The theoretical formulation of Zeta potential is well described by Bhattacharjee^90^. To ensure the accuracy and confirmation of the results in this research zeta potential were performed in two circles.

**Figure 9:**
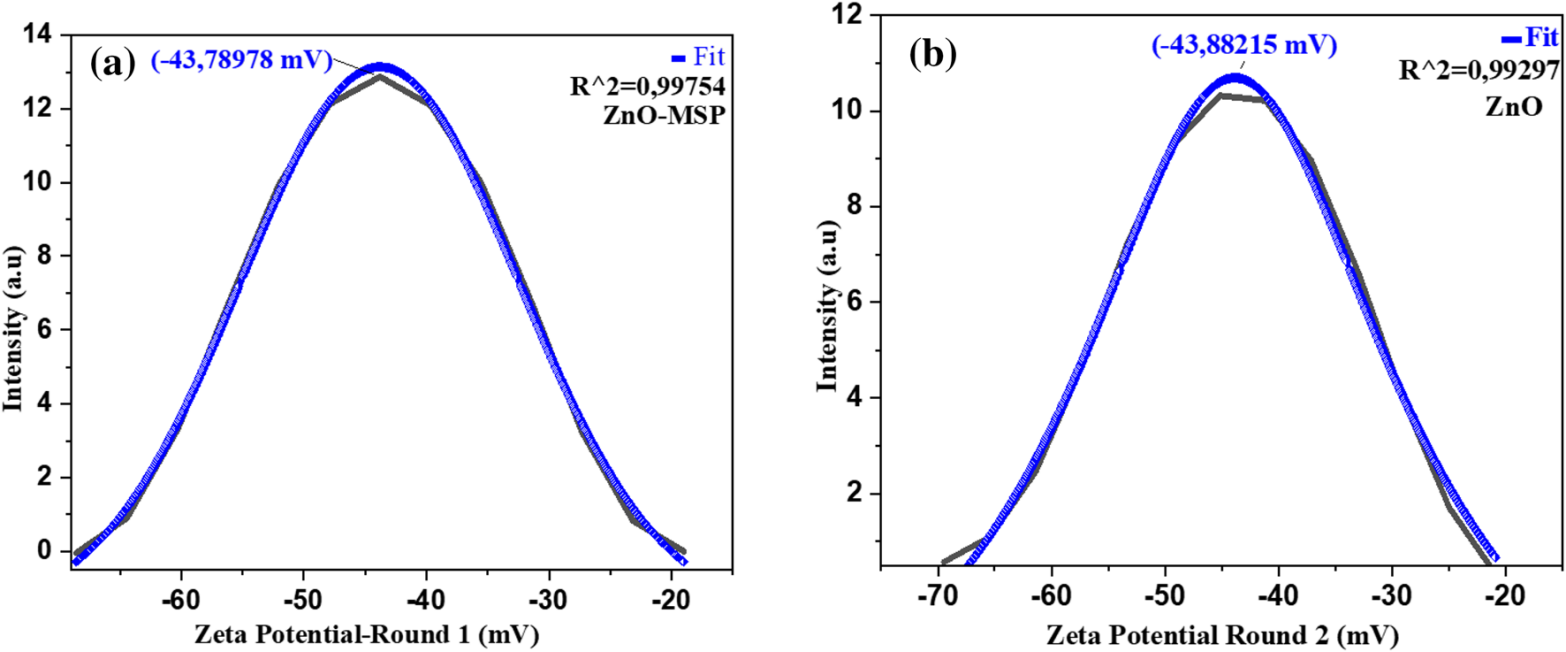
Zeta potential measurement for ZnO colloidal suspension.

The experimental procedure involved mixing 0.2 mg of ZnO-MSP with 20 ml of deionized water (DW pH 7). The mixture was then subjected to probe sonication for 3 min. The zeta potential values obtained for each trial are depicted in Fig. 9. According to the findings reported in reference^55,91^ it was observed that the surface of the particles in the colloidal system carried a negative charge. Despite this negative charge, the particles exhibited good stability ($\mathcalligra{z}\sim -43.8 {\text{mV}})$ in the fluid medium^57,91^.

Based on the findings, these particles of zinc oxide (ZnO) are not subjected to rapid coagulation, aggregation, or flocculation when dispersed in a fluid with properties similar to deionized water^55^. Additionally, from the experimental procedure, the green synthesis approach employed for preparation of ZnO involved low levels of chemicals, making the approach promising and potential applications of these materials as a nanofluid.

### Dielectric properties of the ZnO

As outlined in the methodology (“Synthesis of ZnO NPs, thin films coating and pallets preparation”), the measurement data was collected in the frequency range of (5–80 kHz) and temperature (100–320 K) to analyze the behavior of capacitance, relative permittivity, tangent lose, electric modulus and conductivity of the pallet when subjected to an alternating electric field. In the literature, two methods are described to study the dielectric properties of materials from LCR meter. The first approach, discussed by^92,93^, involves analyzing the complex impedance as a function of frequency, ${\text{tan}}(\delta )$ and temperature. The second method, described in this paper, relies on examining the capacitance as a function of frequency, tangent lose ($\mathrm{tan }\delta )$ and temperature^94,95^ from the data collected.

**Figure 10:**
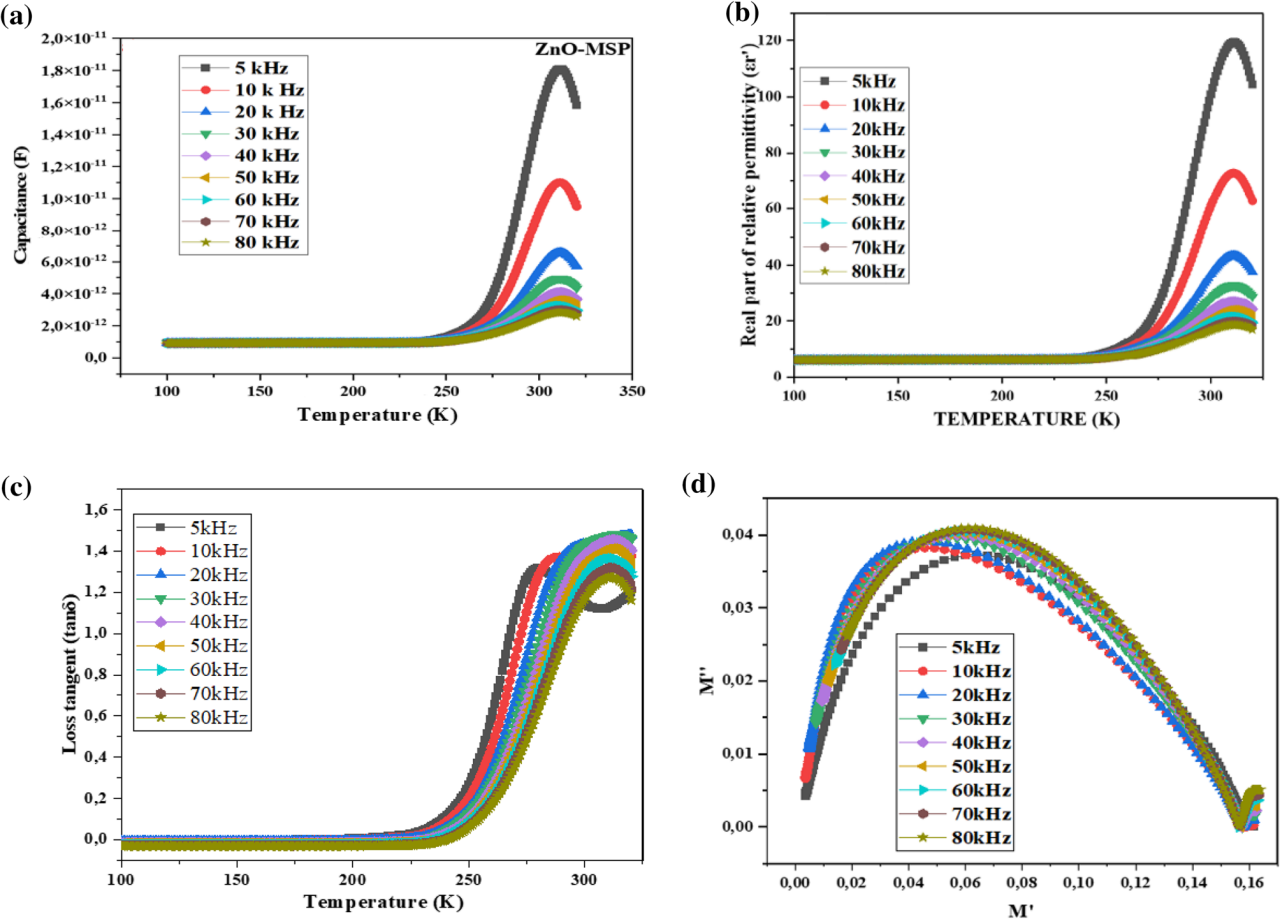
(**a**) Capacitance, (**b**) relative permittivity, (**c**) loss tangent, (**d**) modulus.

The observed decrease in the capacitance (Fig. 10a) as well as the relative permittivity (Fig. 10b) of ZnO with increasing frequency from 5 to 80 kHz can be attributed to various factors including electron scattering, ionic polarization, electrostatic scattering, and dielectric losses. These effects are likely influencing the material's behavior at higher frequencies^93^. However, despite this decrease, it is noteworthy that ZnO exhibited high capacitance values at the frequency of 5 kHz, particularly around room temperature.

To determine the real part of relative permittivity (${\varepsilon {\prime}}_{r})$ of the ZnO, given capacitance values were considered including the shape of the pallet (cylinder-shaped). In the analysis, $C$-capacitance (F), ${\varepsilon }_{r}$ dielectric constant (F/m), $d$ thickness of the pallet (m), $r$ radius ($m$)^96^. The capacitance is given by $C=\frac{{\varepsilon }_{0}\cdot A}{d}$; where K $={\varepsilon {\prime}}_{r}=\frac{C}{{C}_{0}}$. Thus, ${\varvec{\varepsilon}}\boldsymbol{^{\prime}}=\frac{{\varvec{C}}\cdot {\varvec{d}}}{{{\varvec{\varepsilon}}}_{0}\cdot {\varvec{A}}}$; where ${\varepsilon }_{0}=8.85\times 1{0}^{-12} {\text{F}}/{\text{m}}$ $A=\pi \cdot {(\frac{r}{2})}^{2}$, $A=\pi \cdot {(\frac{7.93}{2})}^{2}=49.34 {{\text{mm}}}^{2}$. The imaginary part can be found using ${\varvec{\varepsilon}}\boldsymbol{^{\prime}}\boldsymbol{^{\prime}}={\varvec{\varepsilon}}\boldsymbol{^{\prime}}\times {\varvec{t}}{\varvec{a}}{\varvec{n}}{\varvec{\delta}}$.

A half common surface was used, and the area considered for the analysis was $A=24.68\times 1{0}^{-6}{ {\text{m}}}^{2}$. The real relative permittivity of ZnO, which was eco-friendly synthesized by Moringa seeds, reached a value as high as 120 at a temperature of 310 K and a frequency of 5 kHz. This implies that when an electric field is present, the mentioned ZnO material has a remarkable ability to store electrical energy, nearly 120 times greater than the capacitance of a vacuum. However, at higher frequencies of 80 kHz within the same temperature range, the storage properties of the material were affected by electrostatic scattering, leading to a decrease in the relative permittivity to 18.5.

At low frequencies and within the temperature range of 310 K, ZnO exhibited excellent storage properties, highlighting its potential for utilization as a capacitor or insulator. Additionally, owing to its intrinsic static dielectric behavior, below 250 K, the real part of the relative permittivity (ε′) remained independent of both temperature and frequency^95^.

Figure 10c illustrates the tangent loss (tanδ = $\frac{\varepsilon {\prime}{\prime}}{\varepsilon {\prime}}$) associated with the dissipation of electrical energy when the ZnO pallet was subjected to an alternating electric field. Below 225 K, due to the unique effect of intrinsic static dielectric phenomenon, the levels of loss remained below 2% for all frequencies applied to the material. This suggests that at temperatures below 225 K, the ZnO material exhibited minimal energy dissipation and was characterized by low tangent loss, making it suitable for applications requiring efficient energy storage and minimal losses.

The influence of phase which allow to distinguish between bulk and grain boundaries in nanomaterials is described dielectric modulus by recognizing the relaxation mechanism^93^. ${M}{\prime}=\frac{\varepsilon {\prime}}{{{\varepsilon }{\prime}}^{2}+{\varepsilon {\prime}{\prime}}^{2}}$ and ${M}^{{\prime}{\prime}}=\frac{\varepsilon {\prime}{\prime}}{{{\varepsilon }{\prime}}^{2}+{\varepsilon {\prime}{\prime}}^{2}}$ where M′ is related to storage and M″ to loses dissipative energy in the form of heat. Figure 10d illustrate M″ as function of M′ in the same temperature range given in the Fig. 10c. The peak of relaxation is slightly increasing with frequency which might be attributed to increment of electric field oscillation.

**Figure 11:**
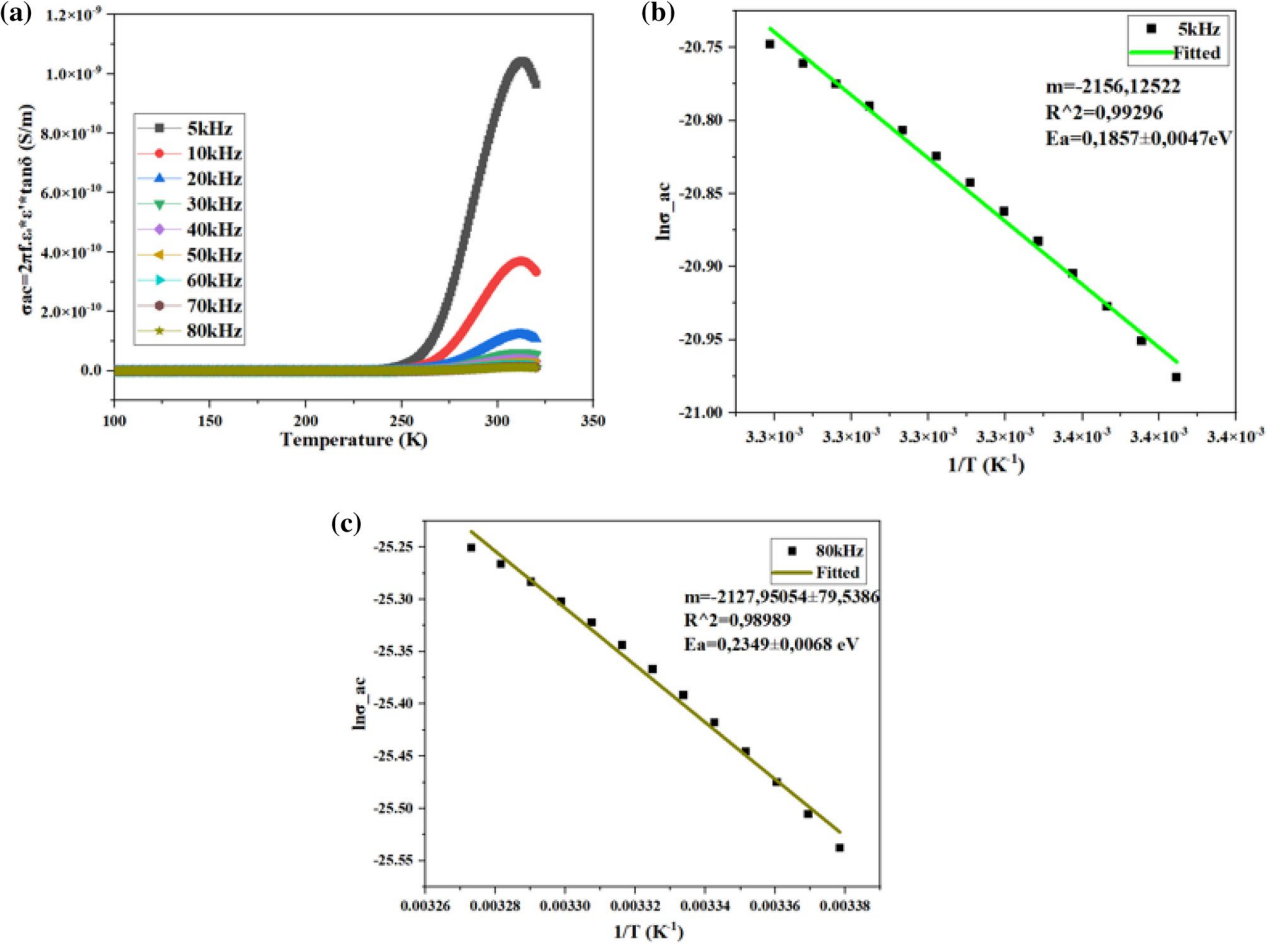
Alternate conductivity (**a**) and energy activation (**b**,**c**).

The alternate current conductivity ${{\varvec{\sigma}}}_{{\boldsymbol{ac}}} \left(\frac{{\varvec{S}}}{{\varvec{m}}}\right)$ was determined by the expression ${{\varvec{\sigma}}}_{{\boldsymbol{ac}}}={\boldsymbol{w}}\cdot {{\varvec{\varepsilon}}}^{{{\prime}}}\times {{\varvec{\varepsilon}}}_{0}\times \textbf{tan}{\varvec{\delta}}$ (Fig. 11a)^97^.

The determination of activation energy ${({\varvec{E}}}_{{\varvec{a}}})$ at room temperature of ZnO pallet was done by Arrhenius law ${{\varvec{\sigma}}}_{{\varvec{d}}{\varvec{c}}}={{\varvec{\sigma}}}_{0}$**×**${{\varvec{e}}}^{-\frac{{{\varvec{E}}}_{{\varvec{a}}}}{{{\varvec{K}}}_{{\varvec{B}}\boldsymbol{*}}{\varvec{T}}}}$, thus **ln** ${{\varvec{\sigma}}}_{{\varvec{d}}{\varvec{c}}}=\textbf{l}\textbf{n}{{\varvec{\sigma}}}_{{\varvec{a}}{\varvec{c}}}-\frac{{{\varvec{E}}}_{{\varvec{a}}}}{{{\varvec{K}}}_{{\varvec{B}}\boldsymbol{*}}{\varvec{T}}}$ where ${{\varvec{K}}}_{{\varvec{B}}}$ **=** $1.30\times 1{0}^{-23}$** JK (**Boltzmann constant). The activation energy is corelated with the slope of Arrhenius functions and Boltzmann constant slope (m) of fitted function is given by ${{\varvec{E}}}_{{\varvec{a}}}=-{\varvec{m}}\cdot {{\varvec{K}}}_{{\varvec{B}}}$. In Fig. 11b,c energy activation is presented for maximum 80 kHz and minimum 5 kHz frequencies applied.

## Conclusions

In this research, ZnO nanostructures were successfully synthesized through an eco-friendly route using moringa seed powder, as evidenced by XRD patterns and XPS elemental analysis. The particles exhibited low levels of aggregation, as observed in the FESEM image, leading to good colloidal behavior observed in the zeta potential measurement.

The band gap determined by UV–VIS Spectroscopy was found to be lower compared to previous research on pure ZnO, indicating good electrical properties. Various Raman modes described in the literature were identified in the samples, with the E_2_H dominant mode characteristic of ZnO.

The ZnO thin films exhibited nano-sized solid-filled grains with surface roughness. Dielectric measurements conducted on the samples revealed elevated capacitance and dielectric constant at room temperature, demonstrated by an inverse correlation with the frequency increase. While the conductivity values were marginally low, the procedure affirmed the successful functionalization of these materials in a non-conditioned environment. These findings carry significant implications for diverse applications of pure ZnO powder, pallets, thin films, and nanofluids eco-friendly by Moringa seeds investigated in this study. Additionally, this methodology, along with the utilization of nanostructures tailored for specific applications, warrants further exploration in subsequent research endeavors.

## Data Availability

https://drive.google.com/drive/u/0/folders/1U5dhHVRn_6ftLQucNK4oEawZ4M9pXl8v.
